# Cancer Clinic Redesign: Opportunities for Resource Optimization

**DOI:** 10.3390/curroncol29060318

**Published:** 2022-05-31

**Authors:** Michael Fung-Kee-Fung, Rachel S. Ozer, Bill Davies, Stephanie Pick, Kate Duke, David J. Stewart, M. Neil Reaume, Marcus Ward, Katelyn Balchin, Robert M. MacRae, Shannon Nelson, Julie Renaud, Dennis Garvin, Suzanne Madore, Jason R. Pantarotto

**Affiliations:** 1 The Ottawa Hospital, Ottawa, ON K1H 8L6, Canada; temepst22@gmail.com (B.D.); spick@toh.ca (S.P.); kduke@toh.ca (K.D.); dstewart@toh.ca (D.J.S.); nreaume@toh.ca (M.N.R.); maward@toh.ca (M.W.); kbalchin@toh.ca (K.B.); rmacrae@toh.ca (R.M.M.); shnelson@toh.ca (S.N.); jurenaud@toh.ca (J.R.); dgarvin@toh.ca (D.G.); smadore@toh.ca (S.M.); jpantarotto@toh.ca (J.R.P.); 2 Division of Gynecologic Oncology, University of Ottawa, Ottawa, ON K1H 8M5, Canada; 3 Department of Medicine, University of Ottawa, Ottawa, ON K1H 8M5, Canada; 4 Division of Radiation Oncology, University of Ottawa, Ottawa, ON K1H 8M5, Canada

**Keywords:** learning health system, ambulatory clinic, block schedule, disease site teams, interdisciplinary care, cancer operations, oncology value stream

## Abstract

Ambulatory cancer centers face a fluctuating patient demand and deploy specialized personnel who have variable availability. This undermines operational stability through the misalignment of resources to patient needs, resulting in overscheduled clinics, budget deficits, and wait times exceeding provincial targets. We describe the deployment of a Learning Health System framework for operational improvements within the entire ambulatory center. Known methods of value stream mapping, operations research and statistical process control were applied to achieve organizational high performance that is data-informed, agile and adaptive. We transitioned from a fixed template model by an individual physician to a caseload management by disease site model that is realigned quarterly. We adapted a block schedule model for the ambulatory oncology clinic to align the regional demand for specialized services with optimized human and physical resources. We demonstrated an improved utilization of clinical space, increased weekly consistency and improved distribution of activity across the workweek. The increased value, represented as the ratio of monthly encounters per nursing worked hours, and the increased percentage of services delivered by full-time nurses were benefits realized in our cancer system. The creation of a data-informed demand capacity model enables the application of predictive analytics and business intelligence tools that will further enhance clinical responsiveness.

## 1. Introduction

Ambulatory cancer centers face a fluctuating patient demand and deploy specialized personnel that have variable availability. This paper describes an operational redesign of a cancer clinic outpatient facility, serving a regional population, to address efficiency, sustainability and stakeholder requirements in an ever-evolving landscape of clinical demand.

The Champlain regional cancer program is anchored by The Ottawa Hospital (TOH), an academic health science center that serves a population of 1.5 million people. The program provides specialized services by cancer disease sites including medical, radiation and surgical oncology clinics, in addition to palliative care and survivorship clinics. Some disease sites provide clinics that are multidisciplinary in scope with patients seeing different types of oncologists with a similar focus in the same physical location. Overall, the ambulatory cancer center provides more than 100,000 clinical encounters annually, including more than 12,000 initial consults.

The challenges of providing cancer care for a population are intensified by struggles with resource utilization and demand/capacity balance at major nodes along the care pathways, such as ambulatory cancer clinics. The goal of optimizing services within these pathways is universal in order to improve patient outcomes and experiences within the allocated physical, human and financial resources [1,2]. Our previous work focused on the integration of lung cancer services from diagnosis to the initiation of treatment. We identified that a unifying approach to these challenges could be achieved using a system level strategy anchored in an iterative learning and design process. The components of this process include curated data, the integration of technology and defined integrated teams [3]. Other practitioners have documented these systems-based approaches together with a learning health system vision as successful in meeting similar challenges [4,5]. The attributes of such a global vision have been documented by the Institute of Medicine and several other bodies [6,7].

### Defining the Problem

Prior to the intervention, our clinic performance was similar to other Ontario academic health science centers [8,9]. Symptoms of a system under stress included budget deficits, overscheduled clinics, high rebooking rates, inconsistency in nursing allocation to patient acuity and wait times for services that exceed provincial targets. Delays in cancer care can have a substantial negative impact on the patient outcome [10], so careful attention to optimizing access to constrained resources is important. Our system lacked flexibility to manage shifting demands due to multiple demographic and therapeutic factors, as well as fluctuating available capacity within provider teams. The previous clinic model was based on defined templated clinic schedules for each provider. Professional responsibilities beyond the ambulatory clinic generated by 50 physicians, including scheduled personal time and unexpected HR issues, totaled >600 provider-weeks annually. The template model did not efficiently repurpose resources from clinics scheduled for unavailable providers and did not effectively and equitably align available resources to evolving, specialized demands.

The complexity of ambulatory clinic environments is an acknowledged challenge to creating efficient operational models [11]. Operations research has focused on the unrealized opportunities in ambulatory scheduling, including block schedules, as well as methods for potential improvements throughout cancer care pathways [12,13,14]. The fundamental constraints of the system were representative of substantial quality dimensions. Physician providers maintain an individual practice for the continuity of care while working across multiple disease sites to provide specialized oncology services within a finite system. Preserving this requires a management approach that maximizes resource utilization, teamwork and alignment between the collective agreements and professional expectations of nurses, physicians and clerks. The role of oncology nursing is profoundly impacted by clinic design, challenging the delivery of specialized care utilizing disease-specific expertise.

Fundamental to the elucidation of the problem, both at the provider and the system level, was the increased understanding of our activity through improved data analysis. This paper applies our systems-based approach to the optimization of the oncology ambulatory clinic and supports the optimization of disease site-based services. The challenge of optimizing central resources (HR, space and finances) to support multiple individual providers, multiple disease site groups and varying temporal and disease-specific demands is a critical step in the redesign of cancer care.

## 2. Methods: Description of the Initiative

To simplify our scheduling operations and recognize the dynamics of both demand and capacity, we adapted and implemented a block schedule approach to clinic scheduling. Block schedule models are widely used to manage allocation of surgical capacity to different surgical specialty teams [15,16,17]. These models have the advantage of allocating resources within an overall budget to specific groups of providers serving a specialized pool of patients. Management of capacity within these specialized service domains can be achieved by facilitating repurposing of unused resources between providers and teams. This paper demonstrates the adaptation of these concepts to a version applicable to an ambulatory oncology clinic.

The new model sets data-informed targets by disease site that predict service demand six months in advance to allow for capacity planning.

### 2.1. Five Key Elements in Block Schedule Design

1. Converted service delivery and provider capacity into common units of disease site service minutes.
2. Reviewed current allocation and identified disparities in resource alignment.
3. Created a quarterly predictive model, including capacity for unexpected demand, to coordinate provider availability within disease sites.
4. Active visibility of future gaps in service or constraints in resources supports dynamic allocation of unused capacity.
5. Development of disease site home-bases that aggregate individual practices with nursing and clerical resources, reinforcing the disease site team approach.

### 2.2. Demand/Capacity Analysis

We categorized clinic demand–capacity concerns at the macro and micro level using established methods of operational analysis [18]. The cancer clinic activity was sorted into disease site groups within each oncology division (medical and radiation). Clinic services delivered (demand) and clinics actually assigned (capacity) were retrospectively reviewed.

### 2.3. Block Schedule and Operational Margin

The intent of the block schedule is to offer planned service delivery capacity to meet service demand. This requires a capacity footprint that is larger than the expected demand due to the various sources of operational and logistics variability and is a typical practice in manufacturing and project management [19]. This margin of additional capacity provides flexibility within defined budget for adjustments required due to unplanned and unpredictable events. The operational margin accommodates the empirical, cumulative uncertainty and is ultimately an arbitrary value. Past delivered services were estimated to represent current demand. This does not account for multiple sources of change in the service demand, however, the rolling, interval nature of a block schedule permits for regular adjustments that are beyond what is accommodated in the operational margin.

## 3. Results

### 3.1. Operational Need for Quarterly Block Schedule for Oncology Clinic

The traditional medical practice model, prior to these changes, was based on annual medical appointment meetings and then a standing weekly template of clinics even though oncologists were expected to be out of the clinic, on average, 12 weeks annually. In this template model, the clinic experienced a constant churn of absent providers and requests for additional clinics. Providers offered limited, urgent coverage to each other’s patients during absences. This model generated constant, high volumes of requests for clinics outside the provider’s usual template, with a high operational complexity and cost of operations.

### 3.2. Removing Barriers to Change: Advanced Access Scheduling

Regaining control of the future clinic calendar was an essential prerequisite for the transition to a dynamic model with quarterly updates. In the template system, the clinical appointment calendar was open for 24 months in the future. This was a barrier to a dynamic scheduling model. An essential preparatory project was the implementation of an advanced access scheduling workflow that discontinued booking any patient appointment more than 90 days in the future [20]. These changes were implemented in a rolling manner across the 50 medical practices. This reduced the rebooking of patient appointments and released individual providers from their fixed templates so that a dynamic model could be implemented.

### 3.3. Pre-Implementation Preparation: Demand Capacity Assessment

In the template model there was no mechanism to assess the current demand for oncology services by disease site and plan the corresponding capacity of specialized personnel. There was no specific linkage between budget allocations and the regional demand for specific services. For example, new treatment options have created a new demand for medical oncology services for melanoma, but our center had not increased our planned clinic capacity for these services. We compared the total clinic services delivered in a one-year period to the total clinic hours assigned to the disease site physician templates. The total annual services compared to the total clinics were 122%, suggesting overscheduled and/or overtime clinics. Since some clinics are cancelled without replacement, the actual rate of the overscheduled or overtime clinic service was even higher. Individual disease site teams had variable degrees of balance between demand and capacity.

### 3.4. Launch of Quarterly Block Schedule

The quarterly block schedule was implemented simultaneously with the launch of an electronic medical record (EMR) in June, 2019. This created a large stress in the center as all personnel learned and adapted to both new systems. The post-launch period was one of adaptation to these large changes. The block schedule was iteratively improved within its quarterly cycles. By the following year, it had activity rebounded above pre-change levels in spite of adaptations to pandemic operations, including increased virtual and telemedicine care.

The block schedule provides a flexible, modular and planned model for clinic operations. Visibility and control of the operation are improved, while the key medical and nursing professional needs are supported (Table 1).

The following features were implemented within the block schedule model and are changes from the template model:

- By creating a specific capacity to meet the regional service demand, the clinic budget became tied directly to patient demand for specific services.
- The clinic budget is not impacted if providers enter or depart. Total services planned remain stable and the provider team can determine staffing strategies.
- Interdisciplinary clinics are planned in advance to desired volume and staffed within a defined envelope.
- Scheduling into the block schedule quarterly provides a structured method to adjust to current conditions in demand and capacity, including personnel issues.
- Creating the capacity to repurpose available clinic shifts supports the expected behavior of providers and enables swapping of clinic capacity within a defined overall budget.
- The model increased consistency of operations, promoting a larger percentage of full-time staff that are well-utilized during all shifts.
- The schedule clusters interdisciplinary teams around groups of patients and promotes timely, responsive, patient-centered care as well as a provider work-life balance.

Clustering the workflow around disease site teams supports the allocation of surge resources to a disease site team and can be deployed to case management for the team pool while providing surge capacity.

### 3.5. Outcome Evaluation

Three intervals of a six-month duration were compared to evaluate the implementation of the block schedule model. The periods were selected to be outside the immediate disruption of our data systems that accompanied the launch of the new EMR in June 2019. The post-evaluation period, beginning in September 2019, coincides with the 2nd and 3rd quarter of the block schedule after optimization, based on the experience of the 1st block. As the clinic operations have known seasonal variability, the evaluation samples are matched periods from September to February in the years 2018–2019 (Pre), 2019–2020 (Post) and 2020–2021 (Pandemic). A comparison of the weekly activity during these periods provides an overall snapshot of total clinical activity in the center (Table 2). The average weekly encounters were not statistically different in the Post period compared to the Pre period, but were significantly increased in the Pandemic period (*t*-Test, *p* > 0.05 and <0.0001, respectively). The total encounters delivered in the six-month period increased 1.6-fold from the Pre to the Pandemic period, while the level of nursing staffing was similar. The coefficient of variation describes the spread of the weekly encounters and was reduced in the Pandemic period compared to the Pre period. When the total weekly encounters are more consistent, it is easier to appropriately staff the clinic and fully utilize all personnel.

**Figure 1:**
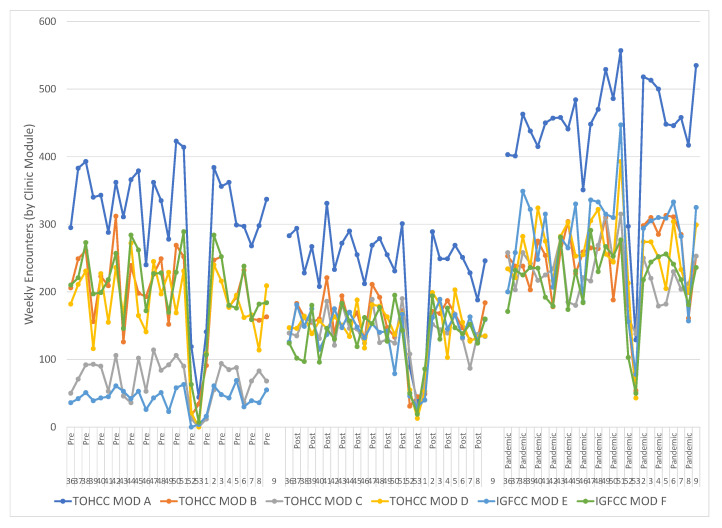
The total weekly activity in the six clinic modules during the three sample intervals (Pre, Post, Pandemic) became less variable and underutilization was reduced. The large activity disruption in each interval is the New Year period. Activity in Mod E was increased from 4% to 17% and in Mod C from 7% to 14% while the fraction in Mod B was reduced from 20% to 15%. Two chi-square tests of independence showed that there was a significant and sustained association between the schedule change and activity distribution in the modules, (Pre-Post *X*^2^ (6, *N* = 25,293) = 4110, *p* < 0.0001, Pre-Pandemic *X*^2^ (6, *N* = 43,431) = 8329, *p* < 0.0001).

The cancer clinics operate out of six physical modules (Mod A–Mod F) containing eight to sixteen exam rooms, located within two sites. Prior to the scheduling change, the activity level varied between modules of similar size as well as within each module week to week (Figure 1). The distribution of activity through all six modules was more uniform after the implementation of the block schedule and the two under-utilized modules were equalized with the rest of the center. Activity in Mod E was increased from 4% to 17% and in Mod C from 7% to 14%, while the percentage of weekly activity in Mod B was reduced from 20% to 15%, comparing the Pre and Pandemic periods. Two chi-square tests of independence showed that there was a significant and sustained association between the schedule change and activity distribution in the modules, (Pre–Post *X*^2^ (6, *N* = 25,293) = 4110, *p* < 0.0001, Pre–Pandemic *X*^2^ (6, *N* = 43,431) = 8329, *p* < 0.0001). These data indicate services are being distributed more evenly through the available clinic capacity and the underutilized capacity has been decreased.

The oscillating variability in the total weekly encounters continued following the schedule change (Figure 1). This variability, generated by multiple known root causes, challenges the quality of care by generating over-staffed and under-staffed clinics. Mitigating this variability requires targeting a consistent volume of clinical activity each weekday alongside consistent staffing targets. This reduces the variability to only those disruptions caused by short-term, last-minute issues from patients, physicians and nurses. A variable volume between weekdays, between busy Tuesdays and slow Fridays, is also undesirable as it forms a barrier to engaging full-time staff and requires more part-time staff.

**Figure 2:**
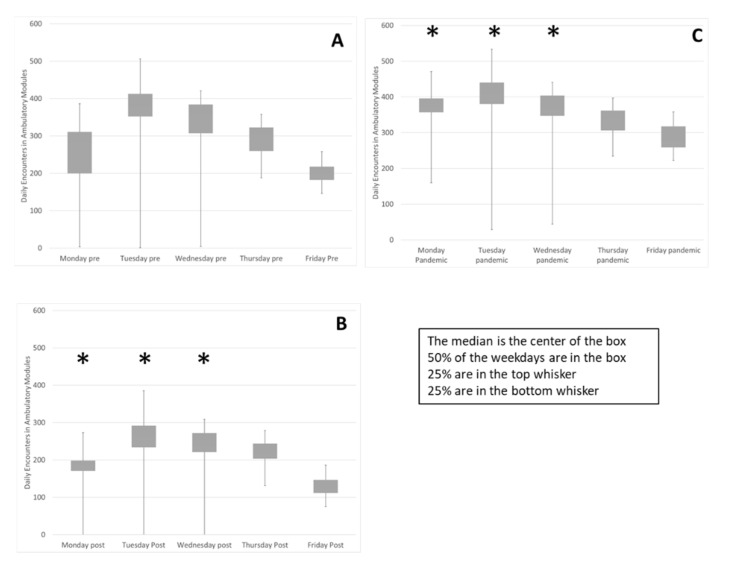
Weekly variability in daily encounters was reduced as well as variability between weekdays. The encounters on each weekday during the three evaluation periods are summarized in box plots (**A**) Pre, (**B**), Post and (**C**), Pandemic. Sustained reduction of variance for Mon, Tues & Wed was observed (*) (*F Test* Pre-Post and Pre-Pandemic *Mon p < 0.0001, p < 0.0001, Tues p < 0.0001, p < 0.0003, Wed p < 0.0001, p < 0.03*). The change on Monday demonstrated by reduction of the coefficient of variation (Standard deviation/mean) from 0.48 to 0.12 (smaller box) and variability decreased on Tuesday and Wednesday due to reduction of low utilization outliers, (smaller bottom whisker). Friday and Monday activity level increased while Tuesdays decreased as proportion of weekly total (flatter row of boxes). Two chi-square tests of independence showed that there was a significant and sustained association between the schedule change and activity distribution between the weekdays, (Pre-Post *X*^2^ (4, *N* = 25,293) = 94, *p* < 0.0001, Pre-Pandemic *X*^2^ (4, *N* = 43,431) = 557, *p* < 0.0001).

Implementation of the block schedule reduced variability within and between weekdays (Figure 2). The median and quartiles for each weekday in the evaluation periods is summarized in a box plot. The interquartile range, contained in the box, trends smaller in the pandemic period, Figure 2C, compared to the Pre state, Figure 2A. The variance of volumes on each weekday was compared between Pre and Post and Pre and Pandemic. Sustained reduction of variance for three weekdays was observed (*F Test* Pre-Post and Pre-Pandemic *Mon p < 0.0001, p < 0.0001, Tues p < 0.0001, p < 0.0003, Wed p < 0.0001, p < 0.03*). The biggest change was on Mondays, reflected in the reduction of the coefficient of variation (Standard deviation/mean) from 0.48 to 0.12 and variability was decreased on Tuesday and Wednesday because the range of low utilization outliers, the size of the first quartile whisker, was reduced.

In the Pre state Figure 2A, Tuesday activity was 26% of the weekly total and Friday activity was 14%. In the Pandemic period Figure 2C, variability between weekdays is reduced. Two chi-square tests of independence showed that there was a significant and sustained association between the schedule change and activity distribution between the weekdays, (Pre-Post *X*^2^ (4, *N* = 25,293) = 94, *p* < 0.0001, Pre-Pandemic *X*^2^ (4, *N* = 43,431) = 557, *p* < 0.0001). Friday activity is increased from 14% to 16% and Monday activity is increased from 16% in the Pre period to 19% in the Pandemic period while Tuesday activity has been reduced from 26% to 24%. These data indicate that utilization of clinic resources is more consistent between weekdays, promoting a consistent workforce.

**Figure 3:**
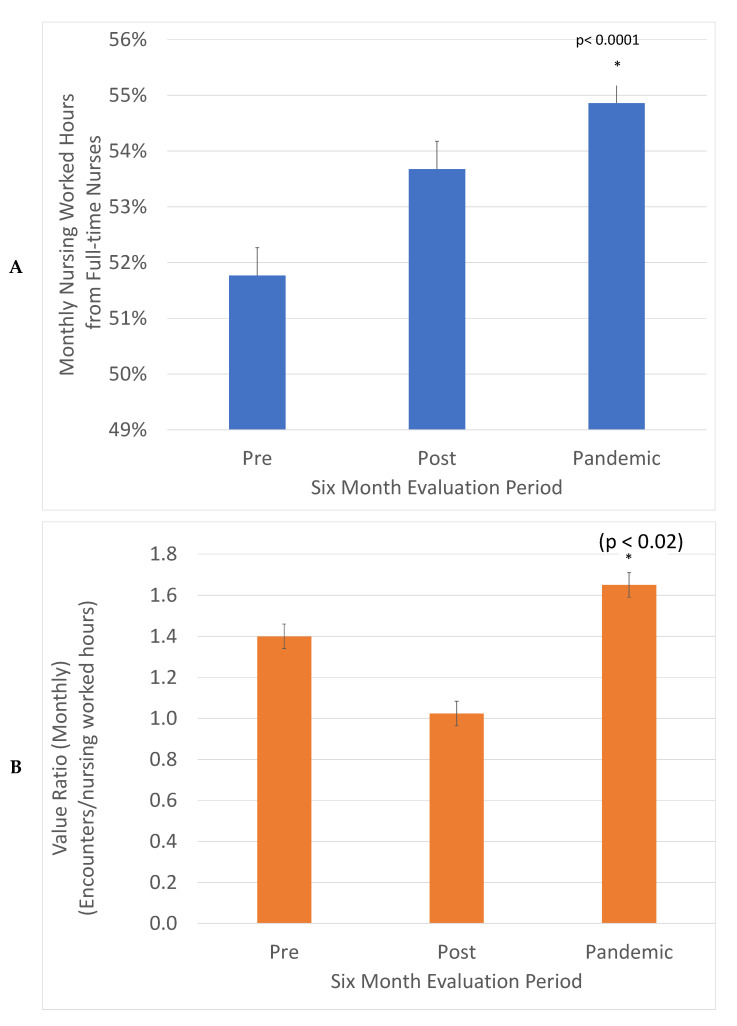
Value (Encounters delivered/Nursing worked hours) was increased 1.17-fold, (*) (*z* test, *p* < 0.02) (**A**) and the percentage of nursing hours from full-time nurses increased from 52% to 55% (*) (*z* test, *p* < 0.0001) (**B**). The total monthly encounters in each evaluation period were divided by monthly worked hours to describe value and the fraction of monthly nursing worked hours from full time nurses.

The value is expressed in healthcare in units of services delivered per costs incurred. This was defined in our project as monthly clinic encounters divided by monthly nursing worked hours (Figure 3). This ratio decreased in the post-implementation period and then increased in the Pandemic period to 1.17-fold higher compared to the Pre period. This was significant in a *z* test, (*p* < 0.02). The increased consistency between weekdays promotes the engagement of full-time nurses. The fraction of nursing worked hours delivered by full-time nurses increased from 52 to 55%, a significant change in the *z* test, (*p* < 0.0001). This is good for patient care as it promotes consistency and expertise in care.

## 4. Discussion

Cancer centers are universally plagued with competing demands for the patient volume, treatment complexity and the allocation of resources within an established budget. In addition, the specialized match of availability between disease-specific providers and patients and the value of continuity of care add profound complexity to the standard challenges of operating a large medical operation. An optimized system must be self-aware (data driven) and adaptable to ongoing variability and challenges or wasteful utilization will creep in. The ability to respond, to learn and to adapt and redesign our processes in a dynamic iterative fashion is an essential component of achieving a truly high-performance cancer service delivery system and is the essential quality of a learning health system [21,22,23,24,25].

The realignment of the cancer center capacity and resources into operational disease site teams is a fundamental step in our journey to integrate disease site clinical pathways and operations across our population, anchored by the physical cancer center. This inherently creates the capacity for us to integrate the continuum of care across screening, diagnostic and surgical groups involved in the multiple disease sites distributed across the region. These premises were the driving force for the initiation of the changes described here.

### 4.1. Operational Excellence through Caseload Management by Disease Site

We have demonstrated that a systems approach and operational performance principles can be applied successfully to a large, multidisciplinary cancer center to address space and provider resource allocations, budget alignment and disease site-specific acuity demands. The block schedule intervention supports caseload management by disease site as it aggregates clinic resources explicitly around the management of distinct patient populations. The designation of nursing and clerical resources to the disease site management increases the clustering of an interdisciplinary care team around similar patients, promoting coordinated, timely care. Enhanced clinical clustering around groups of patients strengthens our ability to learn and provide best-practice care within available resources.

Strategic choices were required to create the solution framework. Consistent with feedback from stakeholders and core principles of oncological care, we identified the following key choices for any future solution:

- Support care continuity for patients and their provider team within the context of disease site group accountability for regional service demand.
- Reformat teams and supporting structures to support this disease site accountability, including nursing allocation and clerical resources.
- Support allocation of resources according to patient acuity by identifying patient segments with different care needs.
- Acknowledge need for quarterly iterative reset to plan human resource staffing and any necessary change to services due to patient demographics or medical science.
- Integrate these concepts into a framework of caseload management by disease site at the individual practice, disease site group and program management levels.

We built an agile and responsive block schedule intervention that was data-informed. This intervention allowed us to accommodate the operational variability that cannot be fully predicted or controlled. The combination of an operational margin and quarterly iteration provides the necessary learning cycles and engagement to manage demand and capacity challenges.

Fundamentally, the ability to distribute clinical activity evenly across the center in response to current demand, within each weekday and across the week, translates into predictable operations which, in turn, allow for the maximizing of physical space and human resources. This creates a measurable increase in value, which we chose to describe with the surrogate ratio of monthly clinical encounters/monthly nursing worked hours.

To demonstrate reduced variability, we documented the improved utilization of the clinical space (Clinic Modules), the smoothed variability for the first three weekdays of the workweek, and the smoothed utilization of the entire workweek by increasing the proportion of clinical work done on Fridays and Mondays. The improved utilization of physical resources included increased proportions of weekly activity in Mod E from 4% to 17% and in Mod C from 7% to 14%, while the fraction in Mod B was reduced from 20% to 15%. A sustained reduction of variance for three weekdays was observed and the biggest change, on Mondays, was reflected in the reduction of the coefficient of variation (Standard deviation/mean) from 0.48 to 0.12. Variability was decreased on Tuesdays and Wednesdays because the range of low utilization outliers was reduced. Smoothing decreased variability across the week with Monday activity increasing from 16% to 19%, Fridays increasing from 14% to 16% and Tuesday activity being reduced from 26% to 24%. While these absolute changes in percentages may seem small, they are operationally and clinically relevant.

The increased value delivered by the intervention was demonstrated by the use of surrogate indicators, the ratio of monthly clinical encounters divided by monthly nursing worked hours. This ratio increased 1.17-fold, demonstrating increased throughput with comparable resources. An additional benefit generated by the cumulative smoothing was the increase of the percentage of nursing services delivered by full-time nurses from 52% to 55%. A consistent service volume between weekdays promotes full-time interprofessional team members, while a variable volume between weekdays requires more part-time and casual personnel that can respond to fluctuating volumes. Predictable and consistent volumes are supportive for a knowledgeable, consistent and experienced clinical workforce that can best support patients.

### 4.2. Change Management

This complex change was made possible through a series of purposeful and discrete steps driven by our overarching systems framework. We built credibility and trust among stakeholders through incremental pilots addressing prerequisites for the initiative. These impacted the role of the clerk, nurse, admin and IT systems. Significant medical and administrative leadership was essential from both formal and informal leadership, including key opinion leaders. Our nursing team reworked the legacy systems to a more team- and disease site-based focus. Our physicians courageously adapted through this change and the simultaneous implementation of the new EMR.

There were a number of challenges during the process of implementation. Coordination of individual practice preferences, nursing perspectives on the optimized professional practice, and system demands for decreased variability and team-based care required intensive communication and adjustments. These dynamic requirements speak to the need for ongoing, iterative redesign which we are now positioned to respond to. The block schedule is comprised of quarterly PDSA cycles and has allowed us to adapt and adjust dynamically. This, together with disease site and divisional team engagement, supports our capacity to adapt to changing practices and HR requirements. The implementation of nursing disease site teams now creates a fertile ground for interdisciplinary quality improvement and operational initiatives across the system.

### 4.3. Adaptability, Pandemic, Virtual Care, Technology, Future Directions

Improved clinic function was demonstrated by the agile response to the COVID pandemic. Our ability to iteratively design our services and align our resources quarterly allowed us to mitigate some of the operational scheduling challenges during the pandemic and rapidly increase and support the provision of virtual care. Caseload management by disease site provided visibility of the clinical demands that were ongoing as the pandemic disrupted our previous service delivery models and, therefore, facilitated a required dynamic adaptation. The implementation of the new EMR improved the pre-planning of clinics and partial charting in advance which was synergistic with the improvements of the block schedule intervention.

Having established a data-driven scheduling system, we are now in a position to deploy interactive dashboards displaying outpatient clinic metrics by disease site and provider. These provide performance feedback to inform the quarterly development of operational targets across groups and individuals. The current optimization supports the ongoing mathematical modeling and incorporation of artificial intelligence and business intelligence tools to reduce the required effort, enhance transparency and equity and further optimize resource utilization.

## 5. Conclusions

The application of a systems vision with a structured improvement framework as a collective project across a whole ambulatory cancer center clinic has resulted in increased agility and efficiency. This supports a dynamic response to service demands while enhancing a disease site group management approach. This approach is predictive, iterative but not prescriptive—the final decision of what is deployed incorporates significant input to account for the nuances of individual physician practice, nurse clinicians, HR needs and disease site service demands. The creation of a data-informed demand capacity model enables the application of predictive analytics and business intelligence tools that will further enhance clinical responsiveness.

## Figures and Tables

**Table 1:** Comparison of Standing Template of Block Schedule Operational Model for Ambulatory Oncology Clinics.

Aspect	Standing Template Model	Block Schedule Model
Physician attendance	Oncologists in clinic 40 weeks/yr, variable mix planned and unplanned absences	Oncologists in clinic 40 weeks/yr, variable mix planned and unplanned absences
Physician planning	Each physician has standing template of weekly clinics and cancels unwanted shifts	Each physician defines expected quarterly schedule in advance within total division footprints
Workload around vacation	Constant churn of physicians requesting makeup clinics before and after absences	Total quarterly clinics planned, aligned to annual goals, then swapped as necessary
Access to consult	Variable capacity depending on template occupancy	Consult clinics reassigned to available providers to maintain access
Workload and cost of staffing	Constant shuffling of nursing and clerical staff between scheduled, cancelled and makeup clinics, vacation/sick calls, unstructured office time	Staffing planned quarterly and shuffling only needed for last minute absences, patient navigation time scheduled and structured.
Physician oversight	Annual physician reappointment meetings to discuss career goals, disease site professional focus areas	Quarterly opportunity to adjust match between demand and capacity, align to annual goals
Budget control, clinic allocation	Budget and space concerns if new providers join team, competition for Tuesdays, slow Fridays	No change to budget or space when new providers join total services aligned to regional demand, more providers can utilize Tuesday and Friday clinics
Alignment demand/ capacity for patient groups	Unclear match rarely updated between regional demand for specialized services and allocated resources	Regional demand for specialized services assessed and allocated as quarterly target for delivery
Interdisciplinary clinics	Difficult to plan consistent interdisciplinary clinics	Clinics allocated sessions of resources and staffed by process within team

**Table 2:** Evaluation of Oncology Clinic Block Schedule Implementation. Weekly encounters are compared in three seasonally matched intervals surrounding implementation of the block schedule in June 2019. A new electronic medical record was also implemented in June 2019.

Parameter	Pre	Post	Pandemic
Interval	September 2018–February 2019	September 2019–February 2020	September 2020–February 2021
Mean	995	937	1670
Standard Error	60	48	65
Median	1019	999	1714
Standard Deviation (SD)	310	250	331
Minimum	89	169	492
Maximum	1339	1167	2265
Sum	26,866	25,294	43,431
*t*-Test for Means, *p* Value compared to Pre period	N/A	>0.05	<0.0001
Coefficient of Variation (SD/mean)	0.31	0.27	0.20

## Data Availability

The data presented in this study is available on request from the corresponding author.
